# Safety of transtympanic application of probiotics in a chinchilla animal model

**DOI:** 10.1186/s40463-017-0242-y

**Published:** 2017-11-22

**Authors:** Carol Nhan, Aren Bezdjian, Shyamali Saha, Satya Prakash, Lily H. P. Nguyen, Sam J. Daniel

**Affiliations:** 1 Department of Otolaryngology – Head & Neck Surgery and McGill Auditory Sciences Laboratory, The Montréal Children’s Hospital, McGill University, 1001 Boulevard Décarie, Montréal, Québec, H4A 3J1 Canada; 2 Department of Experimental Surgery and McGill Auditory Sciences Laboratory, The Montréal Children’s Hospital, McGill University, Montréal, Québec, Canada; 3 Departments of Biomedical Engineering, Physiology, and Artificial Cells and Organs Research Center, Biomedical Technology and Cell Therapy Research Laboratory, McGill University, Montréal, Québec, Canada; 4 Department of Otolaryngology – Head & Neck Surgery and Center for Medical Education, McGill University, Montreal, Québec, Canada

**Keywords:** Probiotic, Otitis, Ototoxicity, Transtympanic, Chinchilla

## Abstract

**Background:**

Chronic suppurative otitis media can be recalcitrant and difficult to treat, particularly with the increasing occurrence of antibiotic resistance. *Lactobacillus plantarum* is a probiotic that has been shown to decrease *S. aureus* and *P. aeruginosa* growth in wounds, making it a good candidate for the treatment of chronic suppurative otitis media. However, before it can be applied in the ear, its ototoxicity potential must be evaluated.

**Methods:**

A prospective controlled trial was conducted in a chinchilla animal model at the Animal care research facilities of the Montreal Children’s Hospital Research Institute to determine whether *Lactobacillus plantarum* is ototoxic when applied transtympanically. Ten chinchillas each had one ear randomly assigned to receive 10^9^ CFU/mL of *Lactobacillus plantarum* solution, while the contralateral ear received saline. Auditory brainstem responses were measured bilaterally at 8, 20, 25 kHz before, at 7–10 days after application, and at 28 days after application of probiotic or saline. Facial nerve and vestibular function were assessed clinically.

**Results:**

There were no statistically significant differences in hearing thresholds between control and experimental ears at 28 days after application. A difference of 11 dB was noted in the 25 kHz range at day 7–10, but resolved by day 28. No animals receiving probiotics developed vestibular nerve dysfunction. There was no histologic evidence of auditory hair cell damaged evidenced by scanning electron microscopy.

**Conclusion:**

Our study suggests that a single application of *Lactobacillus plantarum* at 10^9^ CFU/mL does not cause ototoxicity in a chinchilla animal model. These preliminary safety evaluations and the pathogen inhibitory effects of *L. plantarum* demonstrated by previous studies present this probiotic as a candidate of interest for further investigation.

**Electronic supplementary material:**

The online version of this article (10.1186/s40463-017-0242-y) contains supplementary material, which is available to authorized users.

## Background

Chronic suppurative otitis media (CSOM) can be challenging to treat, particularly when complicated by antibiotic resistance or secondary otomycosis. It is the leading cause of childhood hearing impairment in the developing countries [1] and has had serious implications for speech and language development in children [1–3], impacting cognitive and education outcomes [4]. Serious complications arising from these infections can cause meningitis, intracranial abscess, facial palsy, and lateral sinus thrombosis [5].

The majority of infections are polymicrobial and involve chronic inflammation of the middle ear. Pathogens most commonly associated with CSOM are *Pseudomonas, Staphylococcus, Peptostreptococcus, Fusobacterium, Prevotella*, and *Porphyromonas* [6].

Currently, the first-line treatment for uncomplicated CSOM involves antibiotics and anti-inflammatory agents applied topically to the ear. This treatment best achieves the highest dose delivery with the least secondary effects [7]. However, overuse of antibiotics has resulted in resistant pathogens. Moreover, prolonged use of antibiotics has been associated with development of otomycosis [8].

Probiotics are living microorganisms that can provide beneficial effects [9]. For over a decade, probiotic bacteria have successfully treated infections typically related to gastro-intestinal (GIT) diseases [10]. More recently, several non-GIT applications have been investigated [11]. In children with recurrent otitis media, nasal spray applications of probiotics have been shown to reduce their rates of both infection and middle ear effusion [12]. It is hypothesized that probiotics help restore the native polymicrobial population in the nasopharynx, which typically shows reduced levels of nasopharyngeal commensals in cases of recurrent acute otitis media [13]. These studies bring into question whether topical probiotic bacteria application could also be beneficial in the prevention and treatment of CSOM.

*Lactobacillus plantarum* is a probiotic shown to prevent both *S. aureus* and *P. aeruginosa,* the main organisms found in CSOM [6], from establishing wound infections [14]. It also has antagonistic activity against *Peptostreptococcus* [15], an anaerobic organism commonly found in COSM [6]. In a comparison of three strains of lactobacillus, *Lactobacillus plantarum* was found to have the best inhibitory activity against *S. aureus and P. aeruginosa* [14] making it a good potential candidate for the treatment of CSOM. Nonetheless, the ototoxicity potential of probiotics remains to be established prior to investigating otic applications. Thus, before studying whether *Lactobacillus plantarum* is effective in treating CSOM, its safety when applied topically to the middle ear must be determined.

## Methods

### Study overview

Chinchilla were used to verify the ototoxity of *L. plantarum*. The chinchilla was chosen because it is a well-established animal model for hearing loss studies [16–18]. They also have large tympanic membranes and middle ears similar to humans and a cochlea that is readily dissectible. Each chinchilla had a single application of a solution of probiotic transtympanically to the randomly selected experimental ear and phosphate buffered saline (PBS) to the control ear. Hearing was assessed by auditory brainstem responses (ABR) prior to experimental application of probiotic, then at early and late intervals following application. The animals were euthanized and the cochlear structures were analyzed using scanning electron microscopy (SEM).

### Animal care and ethics

The study received approval by the Animal Care Committee of the McGill University Health Centre Research Institute and was conducted at the McGill Auditory Sciences Laboratory in accordance with the guidelines of the Canadian Council for Animal Care. Ten female chinchillas (*C. laniger*, Ryerson Chinchilla Ranch, OH) were included in the study. Throughout the study, chinchillas were kept in temperature and light controlled rooms with free access to water and commercial food by the animal care research facilities of the Montreal Children’s Hospital Research Institute.

### Sample size

The sample size of seven was calculated setting power at 80% and an alpha of 0.05 to show a difference of 20 dB with a standard deviation of 12.6 dB determined on a pilot study. Ten animals were used to account for the potential of animal loss during the study.

### Hearing evaluation

Hearing evaluations of the chinchilla were performed at three different times: at baseline prior to application of the probiotic bacteria, early (day 7–10) and late (day 28) after application of probiotic. Hearing was tested by ABR on chinchilla anesthetized by 5% Isoflurane and maintained with 3% Isoflurane. Acoustic stimuli of 8000, 20,000, and 25,000 Hz pure tone bursts were presented to the chinchilla through insert earphones starting at 80 dB intensity and decreasing by 5 dB until a threshold was reached.

### Probiotic bacteria preparation

*L. plantarum* ATCC 10241 was plated using MRS agar from an 80% (*v*/v) frozen MRS-glycerol stock. The plate was incubated for 24 h at 37 **°**C with 5% CO_2_ to ensure purity. A single colony from the MRS-agar plate was incubated for 24 h at 37 **°**C in 10 mL of MRS broth. A standard curve was derived using the overnight culture of the bacteria to make a solution of 10^9^ colony-forming-units (CFU)/mL in PBS for transtympanic application. The CFU count of the solution administered was determined again by standard colony counting to ensure accurate dosing. The pH and electrolyte content of the administered solution was verified in order to ensure no confounders in the ototoxicity study.

### Transtympanic application

Each of 10 animals had one ear randomized to receive a single application of probiotic bacteria (experimental), while the contralateral ear received a single application of PBS (control). After anesthetising, a radial incision in the antero-inferior quadrant of the tympanic membrane was made and 0.4–0.7 mL of probiotic solution (until the middle ear was filled) was administered into the middle ear via a soft sterile polyethylene tubing catheter. The same volume of PBS was instilled into the control ears following the same protocol.

### Middle ear examination and histology

Four weeks after application of the probiotic, all animals were euthanized. The middle ears were examined for bony or mucosal changes. The cochleae were dissected and fixed in 4% paraformaldehyde. Post-fixation staining with osmium tetroxide and graded dehydration with 30, 50, 70, 80, 90, and 100% alcohol was performed. Specimens were critical-point dried using Leica CPD 030, mounted, gold plated, and viewed using the Hitachi field emission electron microscopy (Hitachi S4700, Tokyo, Japan).

### Statistical analysis

Early (day 7–10) and late (day 28) shifts in ABR thresholds after application of the probiotics were compared using paired T-test between the experimental and control ears across all three frequencies tested (8, 20, 25 kHz). A *p* value < 0.05 was considered statistically significant.

## Results

### Probiotic preparation and dose selection

Previous studies testing the activity of *Lactobacillus plantarum* against *Staphyloccus aureus* or *Pseudomas aeruginosa* on wounds used concentrations of 10^5^ to 1.5 × 10^8^ CFU/ml [19–21]. Standard colony counting of an aliquot of the probiotic solution used gave a count of 1.5 × 10^9^ CFU/mL. The solution had a neutral pH of 7.0. Na + was 156 mmol/L, K+ 1.7 mmol/L, and Cl- 148 mmol/L.

### Observations for physical signs of toxicity

Three animals had to be euthanized before completion of the experiment due to unrelated illness and were therefore excluded from analysis. The remaining seven animals were in good health until the end of the experiment, maintaining steady weight gain and normal behaviors. Commonly accepted physical signs of ototoxicity are evidence of damage to cochleovestibular nerve, resulting signs of vestibular disturbance such as head tilt or disequilibrium.

### Auditory brainstem response threshold shifts

**Fig. 1:**
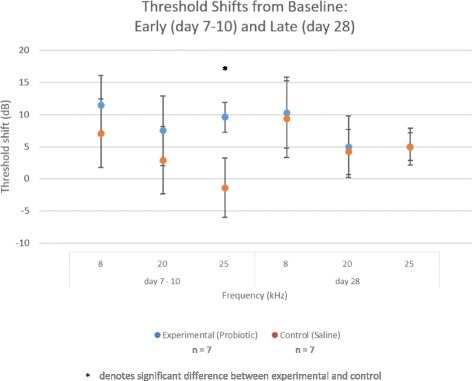
Auditory brainstem response thresholds shifts from baseline. Error bars = 1 standard deviation. Abbreviations: dB, decibels; exp., experimental (probiotic); ctl, control (phosphate buffered saline)

To investigate ototoxicity, baseline hearing measured prior to application of solutions were compared to early post-application (day 7–10) and late post-application (day 28) using ABRs. On the early assessment (day 7–10 following transtympanic application of solution) a significant threshold shift was found at 25 kHz in the ear with the test probiotic doses (9.6 ± 2.3 dB) when compared to the control ear receiving (−1.4 ± 3.5 dB), *p* = 0.02. This threshold shift resolved by the day 28 ABR measurements. No significant long-term hearing loss was observed between experimental and control ears at all frequencies and time points tested (Fig. 1). ABR data per animal is shown in Additional file 1: Table S1.

### Assessment of structural anatomy

Day 1 following application of solutions transtympanically, examination of the tympanic membranes under anesthesia confirmed that the middle ears were still fluid-filled. Prior to measuring early ABRs at day 7–10, ears were again examined otoscopically revealing small amounts of effusions remaining. After euthanasia and temporal bone dissection examination revealed no mucosal changes in the bulla of experimental and control ears.

### Histology

**Fig. 2:**
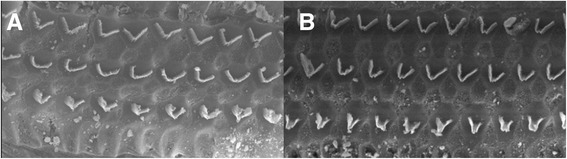
Scanning electron microscopy showing comparison between control (**a**) and experimental (**b**) histological micrographs

Three randomly selected pairs of cochlea were examined under SEM, which revealed no observable changes to the cochlear hair cells between the experimental and control ears for each animal. The three rows of outer hair cells in the Organ of Corti were intact in both the control and experimental ears (Fig. 2).

## Discussion

Treatment of CSOM is problematic particularly when there is antibiotic resistance. Based on studies showing that probiotics can treat various infectious diseases [10–12], the question of whether probiotics could be effective in the treatment of CSOM is raised. Recolonization of the nasopharynx with commensal bacteria has been suggested as a strategy to treat recurrent otitis media [10, 11, 22]. Probiotic bacteria may be a safe and effective adjunct treatment for CSOM.

To date, topical application of probiotic directly to the ear has not been explored. An ideal probiotic would be effective against the pathogens common to CSOM, and have low potential of pathogenicity and ototoxicity. Studies show that *L. plantarum* is active against *P. aeruginosa* and *S. aureus* on wounds [14, 20, 21] and active against methicillin-resistant *S. aureus* in vitro [19], making it a good candidate for treating recalcitrant CSOM. However, the safety of its use in the ear precludes study of its efficacy particularly since some bacteria, such as *S. pneumoniae* and *H. influenzae,* are known to have the potential to cause sensorineural hearing loss through virulence factors [23], bacterial toxins [24], or inflammatory mediators [25]. This explains the association of otitis media with sensorineural hearing loss [26–28].

This study is the first to demonstrate that the probiotic *L. plantarum* at a concentration of 1.5 × 10^9^ CFU/mL applied a single time to the middle ear is not ototoxic as evidenced by ABR results 28 days post-application and by electron microscopy of the cochlea. Such a study is particularly relevant for an organism that is not pro-inflammatory, such as *L. plantarum*, since a non-inflammatory state tends to allow easier permeability through the round window membrane and greater risk of ototoxicity. Since *L. plantarum* has demonstrated ability to limit growth of *P. aeruginosa, S. aureus* [20] and *Peptrostreptococcus* [21], it may be a candidate for further studies investigating its safety and therapeutic use in recalcitrant CSOM.

Inhibition of *P. aeruginosa* growth, as well as inhibition of the production of biofilm and elastase by a 10^5^ CFU/mL solution *L. plantarum* has been demonstrated both in vitro and in vivo [20]. In a burned-mouse model where burn wounds were infected with *P. aeruginosa*, 10^6^ CFU/mL *L. plantarum* applied topically to wounds led to decreased pathogen growth and improved healing. A study of burn patients suggested that topical *L. plantarum* at a concentration of 10^5^ CFU/mL was as effective as silver sulfadiazine (the gold standard in topical burn treatment) in decreasing bacterial load and promotion of wound healing [21].

A 1.5 × 10^9^ CFU/mL solution of *L. plantarum*, a dose greater than that used in the studies on burn wounds, was administered once into the middle ear cavity and confirmed not to cause any toxicity to Chinchilla cochlear structures. The transient statistically significant difference in threshold shift between experimental and control ears seen at 25 kHz during the early (day 7–10) ABR was likely due to the greater viscosity of the probiotic solution compared to PBS. It was noted on physical examination that the experimental ear demonstrated residual effusion at the time of early ABR. Even then this threshold shift was not considered clinically significant at only 11 dB, and most importantly was resolved by day 28.

Although this study serves as preliminary evidence that *L. plantarum* is safe for a single application directly to the middle ear, more complete testing must be done to confirm its safety and effectiveness in humans. Currently *L. plantarum* is widely used in fermented food products including yogurts, as well as probiotic supplements.

Limitations of this study include its small sample size, restricting its ability to detect hearing losses less than 20 dB, and the single application of probiotic rather than multiple applications at intervals, making its findings preliminary in nature. Further investigations evaluating *L. plantarum*’s otologic safety and clinical efficacy are needed. It would also be prudent to study its ototoxicity when used in an animal model of CSOM since that may alter release of cytokines and other factors which may impact ototoxicity. On the other hand, chronic otitis media causes an increase in the thickness of the round window membrane by a factor of two, which could have a protective effect due to decreased permeability of the round window membrane [29–31].

## Conclusion

This study demonstrates that a 1.5 × 10^9^ CFU/mL solution of *L. plantarum* applied a single time intratympanically is not ototoxic in a chinchilla animal model. Based on in vitro, animal, and burn wound studies, this probiotic bacteria could potentially be effective candidate in treating CSOM caused by *P. aeruginosa* and *S. aureus.* Hence, this study’s preliminary otologic safety evaluations and the pathogen inhibitory effects of *L. plantarum* demonstrated by other groups present this probiotic as a candidate for further investigation in treating recalcitrant CSOM. Further pre-clinical and clinical investigations to understand the mechanism of actions responsible for such effects, its safety, and efficacy studies will be invaluable for determining whether *L. plantarum* could be used therapeutically in COSM.

## Additional file

Additional file 1: Table S1. Auditory brainstem response threshold shifts (dB) in the control (phosphate buffered saline) and experimental (probiotic) ears at 7-10 (Early) and 28 (Late) days post application of probiotic. (DOCX 16 kb)

## Abbreviations

**°**C: Degrees celcius
ABR: Auditory brainstem responses
CFU: Colony forming unit
CSOM: Chronic suppurative otitis media
dB: Decibel
Hz: Hertz
kHz: Kilohertz
L: Litre
mL: Millilitre
*P. aeruginosa*: *Pseudomas aeruginosa*
PBS: Phosphate buffered saline
*S. aureus*: *Staphyloccus aureus*
SEM: Scanning electron microscopy
*v*/v: Volume/volume
