# Supplementary material for: Safety of transtympanic application of probiotics in a chinchilla animal model
Source: J Otolaryngol Head Neck Surg. 2017 Nov 22;46:63. doi: 10.1186/s40463-017-0242-y (PMC5700520; doi:10.1186/s40463-017-0242-y)
Supplement: Additional file 1: Table S1. — Auditory brainstem response threshold shifts (dB) in the control (phosphate buffered saline) and experimental (probiotic) ears at 7-10 (Early) and 28 (Late) days post application of probiotic. (DOCX 16 kb) [file 40463_2017_242_MOESM1_ESM.docx]

**Table 1.** Auditory brainstem response threshold shifts (dB) in the control (phosphate buffered saline) and experimental (probiotic) ears at 7-10 (Early) and 28 (Late) days post application of probiotic.

|  |  | **Baseline** | | | **Early (Day 7-10)** | | | **Late (Day 28)** | | |
| --- | --- | --- | --- | --- | --- | --- | --- | --- | --- | --- |
| **Frequency** | **Animal** | **8 kHz** | **20 kHz** | **25 kHz** | **8 kHz** | **20 kHz** | **25 kHz** | **8 kHz** | **20 kHz** | **25 kHz** |
| **Experimental (probiotic)**  **n = 7** | 1 | 30 | 50 | 50 | 40 | 40 | 50 | 30 | 50 | 50 |
|  | 2 | 20 | 20 | 27.5 | 40 | 40 | 40 | **50** | 25 | 27.5 |
|  | 3 | 20 | 30 | 40 | 35 | 40 | 50 | 20 | 30 | 45 |
|  | 4 | 40 | 50 | 50 | 30 | 40 | 55 | 35 | 40 | 50 |
|  | 5 | 30 | 30 | 40 | 60 | 47.5 | 60 | 45 | 40 | 50 |
|  | 6 | 50 | 30 | 50 | 60 | 55 | 60 | 80 | 60 | 65 |
|  | 7 | 30 | 30 | 40 | 35 | 30 | 50 | 35 | 30 | 45 |
| **Control (PBS)**  **n = 7** | 1 | 35 | 40 | 55 | 30 | 40 | 40 | 30 | 40 | 50 |
|  | 2 | 20 | 20 | 30 | 40 | 40 | 40 | 50 | 25 | 30 |
|  | 3 | 30 | 30 | 40 | 30 | 30 | 45 | 25 | 35 | 45 |
|  | 4 | 40 | 50 | 50 | 30 | 40 | 50 | 35 | 40 | 50 |
|  | 5 | 30 | 30 | 40 | 60 | 40 | 55 | 45 | 40 | 55 |
|  | 6 | 50 | 30 | 50 | 60 | 60 | 60 | 80 | 50 | 65 |
|  | 7 | 30 | 30 | 40 | 35 | 40 | 50 | 35 | 30 | 45 |

Abbreviations: dB, decibels; kHz, kilohertz; PBS, phosphate buffered saline.
